# Transmission of malaria and genotypic variability of *Plasmodium falciparum *on the Island of Annobon (Equatorial Guinea)

**DOI:** 10.1186/1475-2875-6-141

**Published:** 2007-10-25

**Authors:** Jorge Cano, Pedro Berzosa, Aida de Lucio, Miguel Angel Descalzo, Leonardo Bobuakasi, Sisinio Nzambo, Melchor Ondo, Jesus N Buatiche, Gloria Nseng, Agustin Benito

**Affiliations:** 1 National Centre for Tropical Medicine and International Health, Instituto de Salud Carlos III, Madrid, Spain; 2 Reference Centre for Endemics Control, Instituto de Salud Carlos III, Republic of Equatorial Guinea; 3 National Malaria Control Programme. Health Ministry and Social Welfare, Malabo, Equatorial Guinea; 4 National Health School, Instituto de Salud Carlos III, Madrid, Spain

## Abstract

**Background:**

Malaria transmission in Equatorial Guinea and its space-time variability has been widely studied, but there is not much information about the transmission of malaria on the small island of Annobon. In 2004, two transversal studies were carried out to establish the malaria transmission pattern on Annobon and analyse the circulating *Plasmodium falciparum *allelic forms.

**Methods:**

A blood sample was taken from the selected children in order to determine *Plasmodium *infection by microscopical examination and by semi-nested multiplex PCR. The diversity of *P. falciparum *circulating alleles was studied on the basis of the genes encoding for the merozoite surface proteins, MSP-1 and MSP-2 of *P. falciparum*.

**Results:**

The crude parasite rate was 17% during the dry season and 60% during the rainy season. The percentage of children sleeping under a bed net was over 80% in the two surveys. During the rainy season, 33.3% of the children surveyed were anaemic at the time of the study. No association was found between the crude parasite rate, the use of bed nets and gender, and anaemia. However, children between five and nine years of age were five times less at risk of being anaemic than those aged less than one year. A total of 28 populations of the three allelic families of the *msp-1 *gene were identified and 39 of the *msp-2 *gene. The variability of circulating allelic populations is significantly higher in the rainy than in the dry season, although the multiplicity of infections is similar in both, 2.2 and 1.9 respectively.

**Conclusion:**

Based on the high degree of geographical isolation of the Annobon population and the apparent marked seasonality of the transmission, it is feasible to believe that malaria can be well controlled from this small African island.

## Background

*Plasmodium falciparum *is a highly polymorphic parasite with a high antigens heterogeneity [1]. This heterogeneity may represent a major obstacle to the development of an effective vaccine [2].

In general, the *P. falciparum *infections include a complex mixture of biologically and genetically different populations, as has been demonstrated by different techniques, including the Restriction Fragment Length Polymorphism (RFLP) [3] and the Polymerase Chain Reaction (PCR) [4]. PCR has been used to study the existing polymorphisms in various markers, such as the Merozoite Surface Proteins 1 (MSP-1) and 2 (MSP-2), the circumsporozoite protein (CSP) and the glutamate-rich protein (GLURP) [5]. Various studies indicate that the genetic diversity in a specific area is related to the level of transmission [5,6]. Therefore, a high prevalence of infection multiplicity has been detected in hyper- and holoendemic zones [7], compared to low transmission zones [8]. Only a number of basal or identical genotypes has been found in low transmission areas, such as Brazil [9] and Honduras [4]. The basis of the genetic diversity is the recombination that occurs in the sexual phase of the parasite within the mosquito. According to this, the higher the transmission, the greater the recombination frequency [10]. However, some authors indicate that the transmission level would not be the only cause for genotype variability [11].

In a study conducted at three locations on the Island of Bioko (Equatorial Guinea), a high allelic diversity has been observed in the *P. falciparum *populations, particularly in the location with the greatest geographical isolation [12]. Furthermore, a correlation was also noted in this study between the parasitaemia levels, age and multiplicity of infection (MOI).

The Annobon population lives in a highly isolated situation, given the distance that separates the island from Bioko and the mainland of Equatorial Guinea. Not much information exists about the transmission of malaria on Annobon. The first known epidemiological and transmission survey dates back to 1987 [13]. During that year, a seroparasitologic study had been conducted among 185 children between two and nine years of age. The crude parasite rate (CPR) was 55.1% and the splenic index of 54.6%. Four years later, another survey was conducted during the dry season, when samples were taken from 1,326 individuals from all age groups, 263 of whom were under five years old. The CPR of children between two to nine years of age was 68%, and 74% in children below five years of age (non published data). In 1993, during the rainy season, the study was focused on 300 children under five years of age. The CPR was 76% in that age group. The parasite density index was 5.8 [14].

No data is available regarding the transmission, even though taking into account the studies conducted on the island of Bioko [15] and on the neighbouring islands of Sao Tomé and Principe [16,17], the malaria vector in Annobon is expected to be *Anopheles gambiae *sensu lato (s.l.).

In 2004 two transversal studies (dry and rainy season) were conducted in order to establish the basics for a control campaign 1) establish the malaria transmission pattern on Annobon, and 2) analyse the circulating *P. falciparum *allelic forms. Finally, a possible eradication of malaria on the island is discussed.

## Methods

### Study area

**Figure 1:**
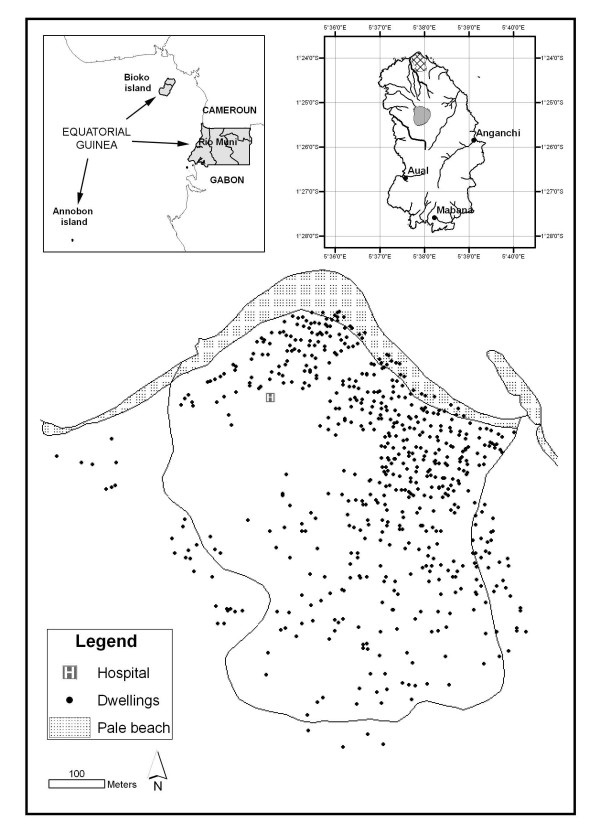
Map of Annobon Island.

Annobon (S 1° 24' W 5° 37') is a small volcanic island (17 km^2^), part of the island region of Equatorial Guinea. It lies to the south of São Tomé and Príncipe, over 300 miles from the Gabonese coastline and just over 600 miles from the country's capital city (Figure 1). It is a rugged island, crossed by a large number of small streams that flow in a radial pattern from A Pot lake, from inland to the coast.

On Annobon, temperature does not fluctuate greatly, with the average temperature being around 26.8°C. The rainy season runs from November to April–May and a dry season from June to October, with an average rainfall of 1,000–1,500 mm/year [13]. Most of the land is dedicated to the cultivation of banana, manioc, plantain, sugar cane and mango, with scarcely any other type of vegetation.

Palé, the urban centre where the most of population lives for the majority of the year, consists of 590 dwellings, inhabited by 2,103 people, 45% of whom are male and 56% female. Thirty-five per cent (706) of the population is under nine years old. The average number of inhabitants per household is 3.59 (0–14, SD = 2.2), and with 1.2 children under nine years old (0–6, SD = 1.3) (2004 population census).

### Data gathering and diagnostic

In 2004, two transversal studies were conducted during the dry (June) and rainy (December) seasons. Sample size in both surveys was calculated according to the prevalence data from seroparasitological studies carried out on 1991 (dry season) and 1993 (rainy season) [13,14]. Sampling design was performed by one-stage cluster sampling. All the children under nine years of age, from a random sample of 100 households selected during each season, were surveyed. The updated population census was used for the scheme and to select the households.

A blood sample was taken from the selected children in order to determine *Plasmodium *infection by microscopical examination of a stained thick and thin film. Each sample was studied by two qualified laboratory technicians. If there was a discrepancy in the result, a third technician was involved in the diagnostics. In addition, the packed cell volume (PCV) percentage was measured and a blood sample was collected on filter paper (Whatman no. 4) for subsequent molecular studies. A dose of sulphadoxine-pyrimethamine was administered to all the children taking part in the study and the use of bed nets was recorded. The adults and legal guardians were asked if the children in question had slept under a mosquito bed net the previous night.

This study had been revised and approved by an ethical committee of the Health and Social Welfare Ministry of Equatorial Guinea and by the National Malaria Control Programme (NMCP). The attendant relative or guardians of the selected children were informed and requested their approval to participate in the studies.

### Diagnostics by semi-nested multiplex PCR and study and the *msp-1 *and *msp-2 *gene allelic diversity of *P. falciparum*

In order to identify and characterize the *Plasmodium *species, a semi-nested multiplex PCR was used, based on amplifying specific ribosomal DNA fragments of the 18S subunit [18].

The diversity of *P. falciparum *circulating alleles was studied on the basis of the genes that encode the merozoite surface proteins, MSP-1 and MSP-2 of *P. falciparum*. In the case of the *msp*-1 gene, the analysis of the three allelic families was carried out: K1, MAD20 and RO33. The variability in the *msp-2 *gene was studied globally and not at the level of the two allelic families described (FC27 and 3D7). The nested PCR, based on amplifying variable zones of the *msp-1 *and *msp-2 *gene, was performed according to the conditions indicated by the authors with some modifications [19].

The amplification product was analysed using the Agilent DNA 7500 Lab Chip^® ^bio-analyser. The number and exact size of the amplification fragments were determined for each sample.

### Statistical analysis

An Access 2000 database was created with the data gathered and the SPSS (v. 12) statistical software was used to analyse the data.

The comparisons between groups were performed using the Pearson and Fisher chi-squared test for categorical variables and T-test for interval variables. The logistic regression techniques were used to calculate the Odds ratio (OR) of the feasible predictors of anaemia, with anaemia being taken as a packed cell volume (PCV) equal or less to 30% [20], and *P. falciparum *multiplicity of infection (MOI). The MOI value, or minimum number of different *P. falciparum *infections present in a sample, corresponds with the highest number of *msp-1 *and *msp-2 *alleles [12]. A p-value < 0.05 was considered to be statistically different.

## Results

The molecular diagnostics was performed on all the blood samples collected in order to assess the quality and reliability of the microscopic diagnostics. In both surveys, the sensitivity and specificity of microscopic diagnostic were high (above 70% in all cases).

In June 2004 (dry season), a total of 112 children under 9 (60 male and 52 females) were surveyed. The mean age for the males was 3.7 (SD = 2.3) years and 3.2 (SD = 2.4) years in the case of the females (t-test, p = 0.298). In December (rainy season), 83 children (46 males and 37 females) were surveyed. In the same way as in the first survey, there were no significant differences in the mean age of males and females (t-test, p = 0.315), 3.8 (SD = 2.6) and 4.4 (SD = 2.7) years, respectively.

The CPR was 17% (19/112) [Confidence Interval 95%: [10%–24%] in the dry season and 60% (50/83) [CI: 49%–71%] in the rainy season. In the first survey, prevalence of infection was significantly higher among males than females (Fisher chi-squared, p = 0.005). This difference was not noted in the second survey (Pearson chi-squared, p = 0.561) (Table 1).

**Table 1:** Crude Parasite Rate and bed nets use by age groups and sex for season

	**Dry season**					**Rainy season**				
	**n**	**CPR (%)**	**CPR CI (95%)**	**BN use %**	**BN use CI (95%)**	**n**	**CPR (%)**	**CPR CI (95%)**	**BN use %**	**BN use CI (95%)**

**≤ de 1 year**	**29**	3	(0–10)	90	(73–98)	**21**	43‡	(21–64)	86	(64–97)
**1–5 years**	**57**	19	(9–30)	85	(73–94)	**28**	54‡	(35–72)	89	(71–98)
**5–9 years**	**26**	27	(10–44)	76	(55–91)	**34**	76‡	(62–91)	75	(57–89)
**Male **	**60**	27‡	(15–38)	77	(66–88)	**46**	63	(49–77)	73	(59–86)
**Female**	**52**	6‡	(0–12)	92	(85–100)	**37**	57	(40–73)	94	(87–100)
**No Bed net #**	**17**	24	(3–44)			**14**	86‡	(67–100)		
**Bed net**	**92**	14	(7–21)			**66**	53‡	(41–65)		

**‡ p-value < 0.05; # Total bed net use was 109 (3 unknown cases) in the dry season and 80 (3 unknown cases) in the rainy season**.

CPR: Crude parasite rate; BN: bed nets; CI: Confidence interval

In addition, the CPR was higher in the 5–9 year age group in the two seasons, although the difference with respect to the other age groups was only significant in the rainy season (Pearson chi-squared, p = 0.031).

In Equatorial Guinea, four species of human *Plasmodium *have been described, although infections by *Plasmodium vivax *are anecdotical [18]. The main species observed in all the infections was *P. falciparum*, 96% (66/69). The mixed infections (*P. falciparum*/*Plasmodium malariae*) diagnosed by microscopy in three percent of infections were not confirmed by PCR. The gametocytic index (percentage of studied population with gametocytes in blood) was 6% (7/112) during the dry and 14% (12/83) in the rainy season.

The percentage of children sleeping under a bed net was over 80% in the two surveys. Furthermore, the number of girls that claimed to sleep under a bed net was significantly higher than the number of boys, both in dry (Fisher chi-squared, p = 0.036) and rainy season (Fisher chi-squared, p = 0.016). Children aged between five and nine were less protected by a bed net, although the difference with respect to the rest of the groups was not significant (Fisher chi-squared, p = 0.396 in the rainy, Fisher chi-square, p = 0.397 in the dry) (Table 1). Data was collected regarding the state and use of the bed nets by the children surveyed.

The prevalence of anaemia during the rainy season is significantly higher than that observed during the dry season (Fisher chi-square, p < 0.001). In the study conducted during the dry season, only 3.1% (3/98) of the children surveyed had a PCV percentage less or equal to 30%. PCV percentage could not be measured in 14 cases. With such a low sample size, the relation between the anaemia and the rest of the measured variables (CPR, use of bed nets, gender and age) could not be analysed. However, during the rainy season, 33.3% of the children surveyed (26/78) were anaemic at the time of the study. PCV percentage could not be measured in five cases.

Table 2 shows the odds ratio for the different measurements with respect to the anaemia. No association was found between the CPR, the use of bed nets and gender, with anaemia. However, significant differences were found between the different age groups (p = 0.016). Children between five and nine years of age were five times less at risk of being anaemic than those below one year of age.

**Table 2:** Feasible factors for suffering from anaemia in the rainy season

		**Rainy season**			
		**N**	**OR**	**95% CI**	***P***

	**Males**	46	1	-	
**Sex**	**Females**	37	0.7	0.2–2.2	0.542

	**under 1 year**	21	1	-	-
	**1 to 5 years**	28	0.7	0.2–2.5	0.615
**Age**	*5 to 9 years	**34**	**0.2**	**0.04–0.7**	**0.016**

**Sleeps under net #**	**No**	14	1	-	-
	**Yes**	66	0.9	0.2–4.1	0.888

**CPR**	**No**	33	1	-	-
	**Yes**	50	1.2	0.4–3.5	0.738

**# Total bed net use was 80 (3 unknown cases)**.

CPR: Crude parasite rate; OR: odds ratio; CI: Confidence interval

A total of 28 populations of the three allelic families of the *msp-1 *gene were identified and 39 of the *msp-2 *gene. Out of these, 17 for the *msp-1 *gene and nine for the *msp-2 *gene were present in the two climatic seasons. Fifty-seven per cent of the MAD20 allelic families of the msp-1 gene were only present in the samples gather during the dry season and 66.7% of those of the *msp-2 *gene only in the rainy season. The only allelic form belonging to the RO33 family of the *msp-1 *gene was observed in the two surveys (Table 3).

**Table 3:** Circulating *P. falciparum *population diversity for the *msp-1 *and *msp-2 *genes

	***msp-1***						***msp-2***	
	**RO33**	**%**	**K1**	**%**	**MAD20**	**%**		**%**
**Total Populations**	1		20		7		39	
**Common populations**	1	**100.0%**	14	**70.0%**	2	**28.6%**	9	**23.1%**
**Only dry populations**	0	0.0%	1	5.0%	4	57.1%	4	10.3%
**Only rainy populations**	0	0.0%	5	25.0%	1	14.3%	26	66.7%

The variability of circulating allelic populations is significantly higher in the rainy than the dry season (Pearson chi-squared, p = 0.03). During the dry season, the number of *msp-1 *gene allelic populations is similar to that registered in the rainy season (22/23), while in the case of the *msp-2 *gene this number nearly triples (13/35).

The multiplicity of infections (MOI) is similar during the dry and rainy season (t-test, p = 0.3670), the MOI average for the dry season is 2.2 (SD = 1.3), while it is 1.9 (SD = 1.0) for the rainy season.

In the case of the samples taken during the rainy season, no association was observed between age, gender, use of bed nets and anaemia, and MOI values over 1. The number of *P. falciparum *positive samples in the dry season was insufficient to carry out this type of analysis.

## Discussion

The transmission of malaria on the Island of Annobon is stable although mainly occurs during the rainy period. The number of diagnosed malaria cases between 2002 and 2003, in the unique health center of the island (the provincial hospital), was 660 (314 per thousand inhabitants) in the dry season and 1,370 (651 per thousand inhabitants) in the rainy season.

Taking into account the results obtained in the transversal studies and the regional hospital registry, it could be hypothesized about a seasonal malaria transmission in the island. This markedly seasonal transmission pattern had not been observed in previous studies [13,14], nor on the neighbouring islands of São Tomé and Príncipe [21], but had been reported in Gabon [22]. During a study conducted in two locations in south-west Gabon, a transmission peak was noted during the rainy period and *A. gambiae *s.l was identified as the malaria vector. It is necessary to perform an entomological longitudinal study on Annobon to identify the main malaria vector to verify the transmission pattern.

In Annobon, a significant difference was observed in the prevalence of infection between sexes (males more than females) during the dry season. Furthermore, the prevalence of infection was higher in children between five and nine years old, but only during the rainy season. The CPR difference between sexes was not observed in studies conducted in other regions of the country, on the island of Bioko [14,23] and mainland region [14], even though differences have been detected between age groups.

The only malaria prevention strategy in Annobon is currently the use of insecticide-treated bed net. There are no distribution and reimpregnation points on the island, which means that all the bed nets come from the country's capital, Malabo. Despite this, the cover reached on the island is high (80% of the children surveyed said that they slept under a bed net), although some differences were noted between sexes (females more than males) and age groups (five to nine years old less than other age groups). This would neither explain the differences found in the prevalence of infection, nor the reason for the seasonal differences. However, it is possible that the sample size calculated to dry season was insufficient to established differences between groups, because of the low CPR found in this season against preliminary data [13].

On the other hand, the percentage of children surveyed with anaemia was significantly higher in the greater transmission period (rainy season). No seasonal differences in the prevalence of anaemia were observed in Bioko, nor were any associations found with variables such as the sex, age, place of residence and use of the bed net [23]. On Annobon, the risk of suffering from anaemia during the rainy season is five times lower in children between five and nine years of age, despite that age group having the greatest infection prevalence. It is known that, in areas of intense transmission, the development of a certain level of immunity due to the frequent contact with the parasite reduces the risk of anaemia and other severe malaria clinical presentations.

With respect to the analysis of the *P. falciparum *population variability, seasonal differences in the variety of circulating populations were observed. When analysing this difference in depth, it was observed that the majority of allelic forms of the RO33 and K1 families of the *msp-1 *gene are present in the two climatic seasons. However, only one third of the allelic forms of the MAD20 family (*msp-1 *gen) and 23% of the allelic forms of the *msp-2 *gene were present in the samples collected in both seasons. Therefore, it must be attributed the greater seasonal variability to the alleles of the *msp-2 *gene. The multiplicity of infection is high and similar in the two seasons and it is not associated with age, sex, the use of the bed net or suffering from anaemia.

In other regions of Africa with a markedly seasonal transmission, such as is the case in Sudan, it has been observed that the diversity of genotypes and multiplicity of infection reduces significantly with the transmission [24]. In the contrary, in Benín, a country where the transmission is perennial, the decrease in transmission does not have a substantial influence on the diversity of *msp-2 *alleles or on the multiplicity of infection [25]. In a recent study conducted in Guadalcanal (Solomon Islands) [26], where the malaria transmission is intense and perennial, with a marked peak in the rainy season, no seasonal changes were observed in the variability of *msp-1 *gene populations. Furthermore, it was discovered that the allelic variability on these islands was lower than that observed in others countries with a lower transmission level.

Few studies performed to determine the seasonal differences in the allelic diversity of the *msp-2 *gene in island zones are available. During a study conducted on São Tomé Island, up to 43 *msp-2 *alleles were identified, although the variability between climatic seasons was not analysed [27].

In the study conducted on Annobon, the influence of transmission intensity on the variability of the circulating parasite populations has been suggested, particularly for the *msp-2 *gene allelic families.

It is known that the genetic recombination occurs during the sexual phase of the biological cycle of the parasite, therefore one of the factors to be taken into account will be the infection rate in the vector. In Equatorial Guinea, the low anopheline density, compared to neighbouring countries [16], is offset by a high infection rate, close to 20% for some anopheline species in the mainland region [15,28]. These high infection rates would increase the number of possible sexual recombinations within the mosquito and, therefore, would favour the circulation of a greater variety of allelic forms, which it could be explained the high variability of the *msp-2 *gene observed in rainy season.

In this way, it is logical to think that the introduction of efficient control measures would result in a reduction in the population heterogeneity of the parasite. In Annobon, 80% of the children studied said that they slept under a bed net. This high cover contrasts with the prevalence of infection and the differences found in terms of gender and age groups. On the other hand, no association was found between the risk of being anaemic and whether or not a bed net was used, which contradict the results obtained in other African countries [29], even for lower cover levels. The high heterogeneity of *P. falciparum *allelic forms on the island, may suggest that the impact of bed net usage on transmission is low.

This low protective efficiency of the bed nets may be due to biting habits of the vector species that are not compatible with this measure of protection, and poor state-of-repair and/or inappropriate use of the bed nets by the population. These are important aspects that must be studied in the future to learn more about the malaria transmission mechanisms on the island.

With respect to the *P. falciparum *allelic diversity in Annobon, it is conceivable that the lack of efficient control measures, a high entomological inoculation rates and crowded households could be related to this high allelic diversity.

## Conclusion

During the second half of the last century, it was believed that malaria could be eradicated. Good results were obtained in controlling the transmission of the disease in some regions of Asia, South America and Central America and on some African islands (Sao Tomé and Príncipe), and its eradication in the majority of the countries of the Mediterranean basin [30]. On the islands of Sao Tomé and Príncipe, the prevalence of the malaria was reduced to 0.95% in 1982 spraying the dwellings with DDT and wide-scale treatment of the population with chloroquine. Disruption of the campaign and spread of choloroquine-resistant *P. falciparum *resulted in a serious epidemic with many deaths [31]. In 1999, a cross-sectional malariological survey carried out in Príncipe suggested that a good clinical management of malaria cases and the use of preventive measures against mosquitoes bites can probably eliminate endemic malaria [21].

The most recent experience to control and eradicate malaria was in Vanuatu, an island complex in the south-west Pacífic [32]. In 1991, a campaign was set up in Vanuatu, based on the use of bed nets impregnated with permethrin, widespread treatment of the population during a nine-week period and the use of larvivorous fish. After nine years of monitoring the malariometric indicators and controlling for imported malaria, the disease is considered to have been eliminated from some islands in this region of the Pacific.

Taking this experience into account, the high degree of geographical isolation of the Annobon population and the suggested seasonal nature of the transmission, it is conceivable that malaria could be eradicated in this small African island, as long as the sustainability of the malaria control programme can be ensured.

## Authors' contributions

JC was involved in the design of the survey, participated in the data collection and the interpretation of statistical analysis, and coordinated the draft of the manuscript. PB was involved in the molecular studies and helped to draft the manuscript. AL was involved in the molecular analysis. MD was performed the statistical analysis and interpretation, and drafted the manuscript. SN, LB, MO and JB participated in the collection of the data and blood samples. AB and GN participated in the design of the surveys and have given approval of the version to be published. All authors read and approved the final manuscript.
